# Association between sleep quality and dry eye disease: a literature review and meta-analysis

**DOI:** 10.1186/s12886-024-03416-7

**Published:** 2024-04-05

**Authors:** Yixuan Gu, Kai Cao, Ao Li, Jingyi Wang, Yihan Guo, Yiran Hao, Lei Tian, Ying Jie

**Affiliations:** 1 Beijing Institute of Ophthalmology, Beijing Tongren Hospital, Beijing Ophthalmology & Visual Sciences Key Laboratory, Beijing Tongren Eye Center, Capital Medical University, No. 1 Dong Jiao Min Xiang, Dong Cheng District, 100730 Beijing, China; 2 Beijing Institute of Ophthalmology, Beijing Tongren Hospital, Beijing Ophthalmology & Visual Sciences Key Laboratory, Capital Medical University, 100730 Beijing, China

**Keywords:** Dry eye, Sleep quality, Sleep disorder, Meta-analysis

## Abstract

**Objective:**

The purpose of this article is to systematically review the association between dry eye and sleep quality.

**Methods:**

PubMed, EMBASE, Cochrane, Web of Science, and grey literature databases were searched for observational studies published before April 2023. Meta-analysis was performed using STAT15 software.

**Results:**

A total of 21 studies with 419,218 participants were included. The results showed that the dry eye subjects had a worse sleep quality than the healthy population, with poorer subjective sleep quality, longer sleep latency, and a higher risk of unhealthy sleep duration such as insufficient sleep or excessive sleep. The Pittsburgh Sleep Quality Index (PSQI) scores of the dry eye subjects were significantly higher than those of the control subjects (WMD = 1.78, 95%CI: 1.06, 2.50, *P* < 0.001). The dry eye subjects scored higher than the control subjects in sleep quality, sleep latency, and sleep disturbance in PSQI; there was no difference between the dry eye individuals and control subjects in sleep duration, sleep efficiency, daytime dysfunction, and sleep medication scores. The risk of sleep disorders in the dry eye subjects was significantly higher than that in the non-dry eye subjects (RR = 2.20, 95%CI: 1.78, 2.72, *P* < 0.001); the risk of insufficient sleep in the dry eye subjects was higher than that in the control subjects (RR = 3.76, 95%CI: 3.15, 4.48, *P* < 0.001), and the prevalence of excessive sleepiness in dry eye subjects was higher than that in the control subjects (RR = 5.53, 95%CI: 3.83, 7.18, *P* < 0.001). The ESS scores of the dry eye subjects were significantly higher than those of the control subjects (WMD = 3.02, 95%CI: 2.43, 3.60, *P* < 0.01).

**Conclusion:**

Our meta-analysis suggests that individuals with dry eye have a worse sleep quality than the healthy population, with poorer subjective sleep quality, longer sleep latency, and higher risk of unhealthy sleep duration such as insufficient sleep or excessive sleepiness.

**Supplementary Information:**

The online version contains supplementary material available at 10.1186/s12886-024-03416-7.

## Introduction

Sleep is an essential physiological process for life, accounting for approximately one-third of human daily activities. In recent years, bad sleep quality, including sleep disorder, insomnia, circadian rhythm disorders, and excessive sleep, have become a persistent global social issue [1]. Previous studies have shown that sleep disorder was associated with higher risks of hypertension [2], diabetes [3], cancer [4], and other adverse outcomes [5]. Although pharmacological therapies have applied to the management of sleep disorder, chance to receive sleep therapy is limited owing to the lack of sleep therapists and sleep clinics [6]. Due to lifestyle changes and the dramatic increase in the population with sleep disorders, identifying potentially modifiable risk factors is clinically important for the primary prevention of sleep disorders.

Dry eye is a multifactorial ocular surface disease characterized by an imbalance in tear film homeostasis, accompanied by varying degrees of tear film instability, increased osmotic pressure, inflammation, and nerve damage [7]. Poor sleep quality in dry eye patients may be related to incomplete eyelid closure, eye discomfort, and mental stress [8]. The dry mouth symptoms of Sjogren’s syndrome patients, which require them to frequently get up at night to drink water or consume more fluids before sleep, may also be one of the culprits causing poor sleep quality. Accordingly, there are emerging studies that have indicated that dry eye was associated with the risk of sleep disorder [9, 10]. A cross-sectional study by Kawashima et al. [9] including 672 participants aged 26–64 years from Osaka reported that sleep quality measured by Pittsburgh Sleep Quality Index(PSQI) score in dry eye patients was significantly poorer than that in non-dry eye individuals. However, only one meta-analysis suggested that participants with dry eye had poorer sleep quality, more daytime sleep, shorter total sleep duration, and higher prevalence, incidence, and severity of sleep disorders compared to participants without dry eye [10].However, present studies were limited by the small sample size included, years only up to 2018, and the inaccurate selection criteria for the dry eye subjects.

Therefore, the purpose of the current study was to systematically review the association between dry eye and sleep disorder by performing a meta-analysis of the evidence across existing observational studies to scientific evidence for the prevention of sleep disorders.

## Method

### Literature search strategy

A comprehensive search was conducted in PubMed, EMBASE, Cochrane, Web of Science, and gray literature databases, and appropriate search strategies were developed using “dry eye” and “sleep disorder” as search targets to retrieve articles as comprehensively as possible (see Annex). The search was conducted up to April 2023. To ensure the comprehensiveness of the retrieved literature, no restrictions were placed on the publication date, location, or ethnicity. See Supplementary material 1 for specific search strategies.

### Inclusion and exclusion criteria

The inclusion criteria for this study were: (1) subjects aged ≥ 18 years; (2) case subjects diagnosed with dry eye or Sjogren’s syndrome, with diagnostic criteria provided, and the control subjects consisting of healthy individuals; (3) provision of the number of bad sleep quality or sleep scores for both case and control subjectss; (4) study type is observational research.

Exclusion criteria: (1) inappropriate article types, such as reviews, meta-analyses, conference abstracts, case reports, etc.; (2) animal experiments; (3) unobtainable data or inappropriate data types; (4) duplicate publications or articles without full-text availability; (5) control subjects with other eye diseases.

### Data extraction

Two researchers independently screened the articles based on titles and abstracts, and then further screened the full texts according to the inclusion and exclusion criteria. In case of disagreement, a third researcher was consulted for resolution. Information extracted included author(s), country, study type, data collection period, publication year, type of dry eye, number of participants in the dry eye and control subjectss, age, female proportion, and sleep-related judgment criteria.

### Quality assessment

Quality assessments were conducted for the finally included articles based on their types. For cohort and case-control studies, the Newcastle-Ottawa Scale (NOS) was used to [11, 12] assess the selection of cases and controls/cohorts, comparability, exposure and outcome. The assessment included appropriateness and representativeness of case or cohort selection, determination and reliability of exposure, comparability of cases or cohorts, response rate or follow-up rates and duration, consistency of investigation and outcome measurement methods, etc., totaling 9 points, with 2 points for “comparability”. The final scores were categorized as: 0–3 points = low quality; 4–6 points = medium quality; 7–9 points = high quality [13]. For cross-sectional studies, the Agency for Healthcare Research and Quality (AHRQ) scale was used for evaluation, with a total of 11 items and 11 points, covering the definition of information sources, inclusion and exclusion criteria, time frame and continuity of patient identification, subjective factors of evaluators, quality assurance assessment, confounding and missing data, and patient response rate and completeness. For each question in the questionnaire, 1 point was awarded for a “yes” answer, and no points were awarded for a “no” or “unclear” answer. The final scores were related to the article quality as follows: low quality = 0–3; medium quality = 4–7; high quality = 8–11 [13].

### Statistical method

Data were analyzed using the STATA15 software. Continuous variables were expressed as weighted mean difference (WMD), and non-continuous variables were reported as relative risk (RR). Both were provided with 95% confidence intervals (CI), and forest plots were drawn. Heterogeneity between studies was assessed using the Q test and I^2^ test: if the test result was *P* ≥ 0.10 and I^2^ < 50%, homogeneity among studies was assumed, and a fixed-effects model was chosen; if *P* < 0.10 and I^2^ ≥ 50%, heterogeneity was considered among the included studies, and a random-effects model was chosen. For outcome measures reported in more than 10 included articles, Egger’s test was used to assess potential publication bias. Sensitivity analysis was performed by sequentially excluding individual articles. If the results obtained after excluding individual articles did not show significant deviation, the results were considered relatively accurate. A *P*-value of < 0.05 was considered statistically significant.

## Result

### Literature screening process and results

**Fig. 1:**
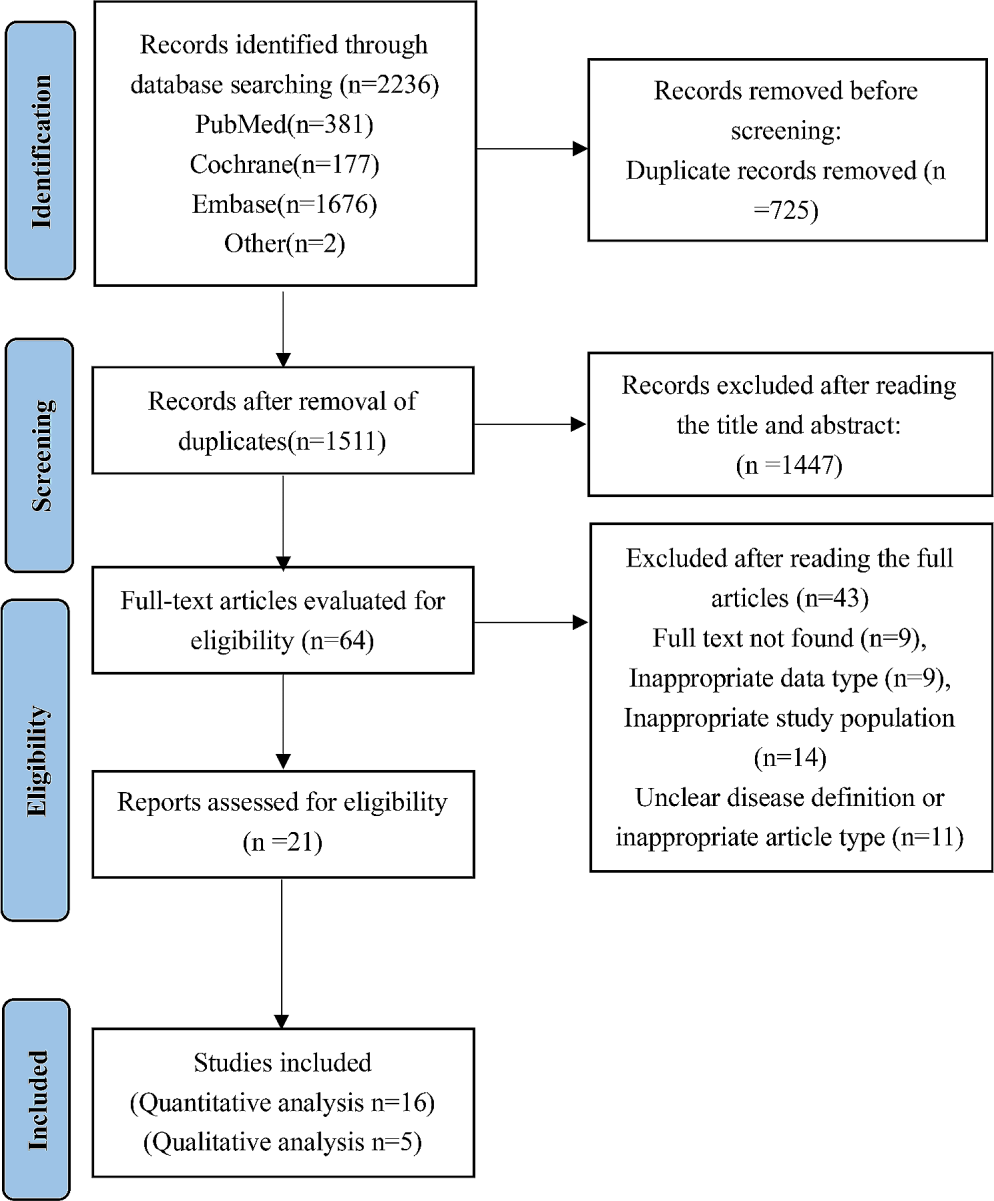
Flow chart of literature search

A total of 2236 related articles were retrieved, and after deduplication, 1511 articles remained. By reading the titles and abstracts, 64 articles remained after excluding those that did not meet the inclusion and exclusion criteria. After reading the full articles, 43 articles were excluded, including 9 articles with unavailable full texts, 9 articles with unsuitable data types, 14 articles with unsuitable study subjects, and 11 articles with unclear disease definitions or unsuitable article types. A total of 21 articles were finally included in this study [8, 9, 14–32]. The literature screening process and results are shown in Fig. 1.

### Study characteristics

This study included a total of 419,218 patients, with 152,567 patients in the dry eye subjects and 266,651 patients in the healthy control subjects. The study characteristics are shown in Table 1, with all articles being of medium to high quality. Among them, there were 12 cross-sectional studies [8, 9, 14–18, 21, 22, 25–27], 6 cohort studies [19, 23, 29, 31, 32], and 3 case-control studies [24, 28, 30]. The studies involved cases from the United States (1) [17], Australia (1) [28], China (3) [30–32], India (1) [24], Italy (1) [26], Japan (3) [9, 14, 27], South Korea (5) [8, 16, 21, 25, 31], the Netherlands (2) [23, 29], Singapore (1) [22], Sweden (1) [18], Turkey (1) [15], and the United Kingdom (1) [19]. There were 14 studies with a dry eye-only experimental subjects [8, 9, 14, 17, 21–25, 27, 30–32] and 7 studies with a Sjögren’s syndrome subjects [15, 16, 18, 19, 26, 28, 29]. The judgment of sleep disorder was based on the PSQI questionnaire (including the CPSQI questionnaire) in 8 studies [9, 14–16, 23, 26, 30, 31], the ESS questionnaire in 5 studies [15, 16, 19, 22, 28], the ISI in 2 studies [17, 22], the IRLS in 1 study [15], the ICD-10 disease code in 1 study [32], other self-assessment questionnaires in 4 studies [8, 21, 24, 27], the 15-item Dutch questionnaire on sleep quality in 1 study [29], the Uppsala Sleep Inventory in 1 study [18], and polysomnography in 1 study [28].

**Table 1:** Basic characteristics

Author	Years	Country	Study type	Data collection period	DE			Control subjects			Female Proportion	Sleep-related judgment criteria
					Type	Number of participants	Age	Female Proportion	Number of participants	Age		
Zheng	2022	China	Cohort	2013–2020	DE	128,966	> 18	35.01%	128,966	> 18	64.99%	ICD-10: G47
Gudbjörnsson	1993	Sweden	CS	-	pSS	40	53 ± 12	95%	60	53	100%	Uppsala SleepInventory
Usmani	2012	Australia	CC	-	pSS	28	58.7 ± 1.9	100%	18	55.8 ± 3.4	100%	ESS, Polysomnography
Ayaki	2016	Japan	CS	2014–2016	DE	106	53.2 ± 10.7*	100%	107	48.3 ± 11.4	100%	PSQI
Kawashima	2016	Japan	CS	2011	DE	249	42.4 ± 8.1	40.16%	134	42.7 ± 9.0	23.13%	PSQI
Lee	2015	South Korea	CS	2010–2012	DE	2835	> 20	72.77%	13,043	> 20	52.72%	self-report questionnaire
Galor	2018	United States	CS	2013–2016	DE	153	62.36 ± 9.35	0.8%	34	63 ± 11	6%	ISI
Yu	2019	China	Cohort	2016–2017	DE	1126	53.53 ± 14.05	59.15%	1704	48.8 ± 13.5	53.3%	CPSQI
Nithish Raj	2020	India	CC	2022	DE	75	20–50	-	75	20–50	-	self-report questionnaire
Magno	2021	The Netherlands	Cohort	2006–2013	DE	6414	19–94	77.72%	65,347	19–94	57.57%	PSQI
An	2022	South Korea	CS	2010–2012	DE	2940	≥ 20	72.89%	13,531	≥ 20	54.62%	self-report questionnaire
Park	2021	South Korea	CS	2016–2018	DE	602	≥ 40	79.07%	1904	≥ 40	59.98%	-
Takahashi	2020	Japan	CS	2016–2016	DE	890	44.0 ± 13.8	59.66%	1110	45.2 ± 13.9	42.25%	self-report questionnaire
Lim	2019	Singapore	CS	2011–2015	DE	265	64.31 ± 10.09	47.17%	3038	63.24 ± 9.64	53%	ISI ESS
Wu	2019	China	CC	-	DE	106	45.52 ± 12.8	80.2%	50	43.1 ± 12.32	76%	PSQI
Han	2019	South Korea	Cohort	2022 − 2015	DE	7429	> 18	65.49%	36,937	> 18	53.08%	-
van Oers	2010	The Netherlands	Cohort	-	pSS	29	53.3 ± 13.8	100%	52	51.2 ± 12.1	100%	15-itemDutch questionnaire on sleep quality
Hackett	2012	UK	Cohort	2010	pSS	69	60 ± 13	93%	69	62 ± 11	93%	ESS
Cho	2015	South Korea	CS	2014–2015	pSS	139	45.22 ± 11	69.8%	363	41.76 ± 11.55	60.9%	PSQI ESS
Priori	2015	Italy	CS	-	pSS	29	55.58 ± 11.95	100%	29	55.67 ± 12.07	100%	PSQI
Balkarli	2016	Turkey	CS	-	pSS	77	46 ± 10.5	90.9%	80	44.7 ± 9.3	87.5%	PSQI ESS IRLS

*Errors in the author’s table

DE, dry eye; CC, case control; CS, cross sectional; ICD-9-CM: International Classification of Diseases, 9th ed, Clinical Modification; ESS, Epworth Sleepiness Scale; PSQI, Pittsburgh Sleep Quality Index; CPSQI, Chinese Pittsburgh Sleep Quality Index; pSS, primary Sjogren’s syndrome; ISI, Insomnia Severity Index); IRLS, International Restless Leg Scale

Quality assessment of the included literature showed that the cross-sectional study quality scores ranged from 6 to 10, indicating that the included studies were of medium to high quality. Cohort studies scored 8–9 points, representing high-quality articles. Case-control studies scored 6–8 points, representing high-quality articles.

### Total PSQI score

**Fig. 2:**
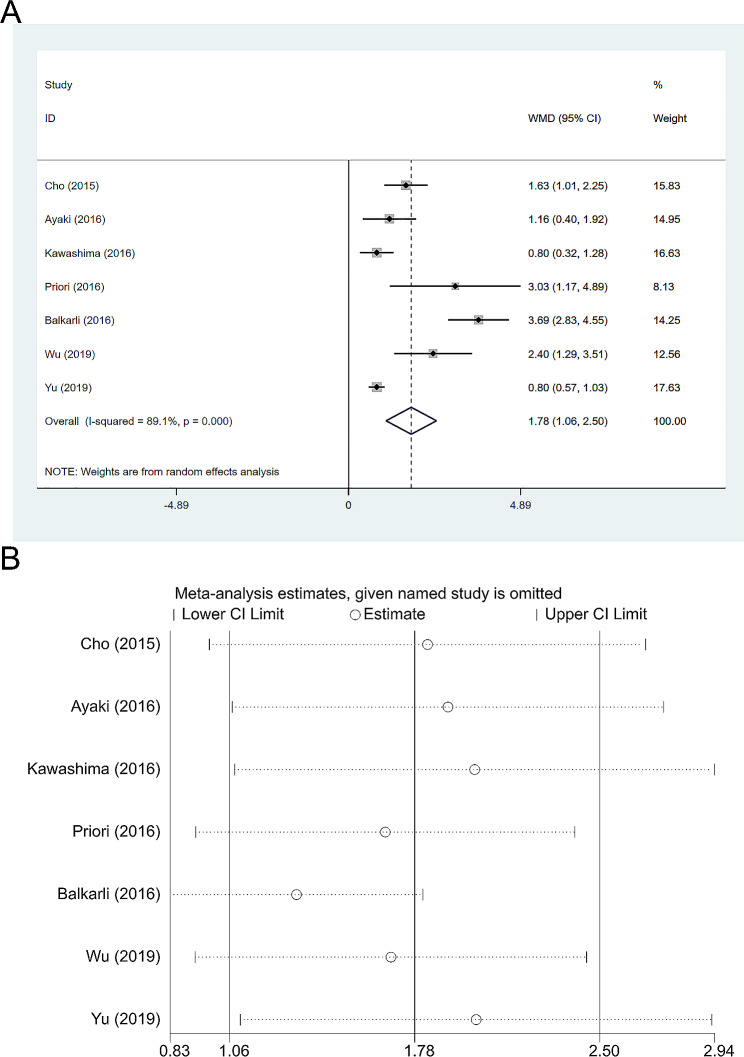
**A**: PSQI total score; **B**:Sensitivity analysis of PSQI total score

The PSQI (Pittsburgh Sleep Quality Index) is a widely used and recognized questionnaire for assessing sleep quality [1], which is subjectively completed by the patients. The higher the score, the worse the sleep quality. In the included articles, 7 articles [9, 14–16, 26, 30, 31] mentioned the PSQI total score and used it to evaluate the sleep quality of the patients. There was significant heterogeneity between the studies (I^2^ = 89.1%, *P* < 0.001), so a random-effects model was used (see Fig. 2A). It was found that the PSQI score of the dry eye subjects was significantly higher than that of the control subjects (WMD = 1.78, 95%CI: 1.06, 2.50, *P* < 0.001), indicating that the sleep quality of dry eye patients was worse than that of healthy people, and this difference was statistically significant. A sensitivity analysis was conducted on the included articles for this indicator (Fig. 2B), and it was found that the exclusion of individual articles did not significantly affect the main results. At the same time, since the number of included articles was less than 10, Egger’s test was not performed.

### PSQI Subitem scores

**Fig. 3:**
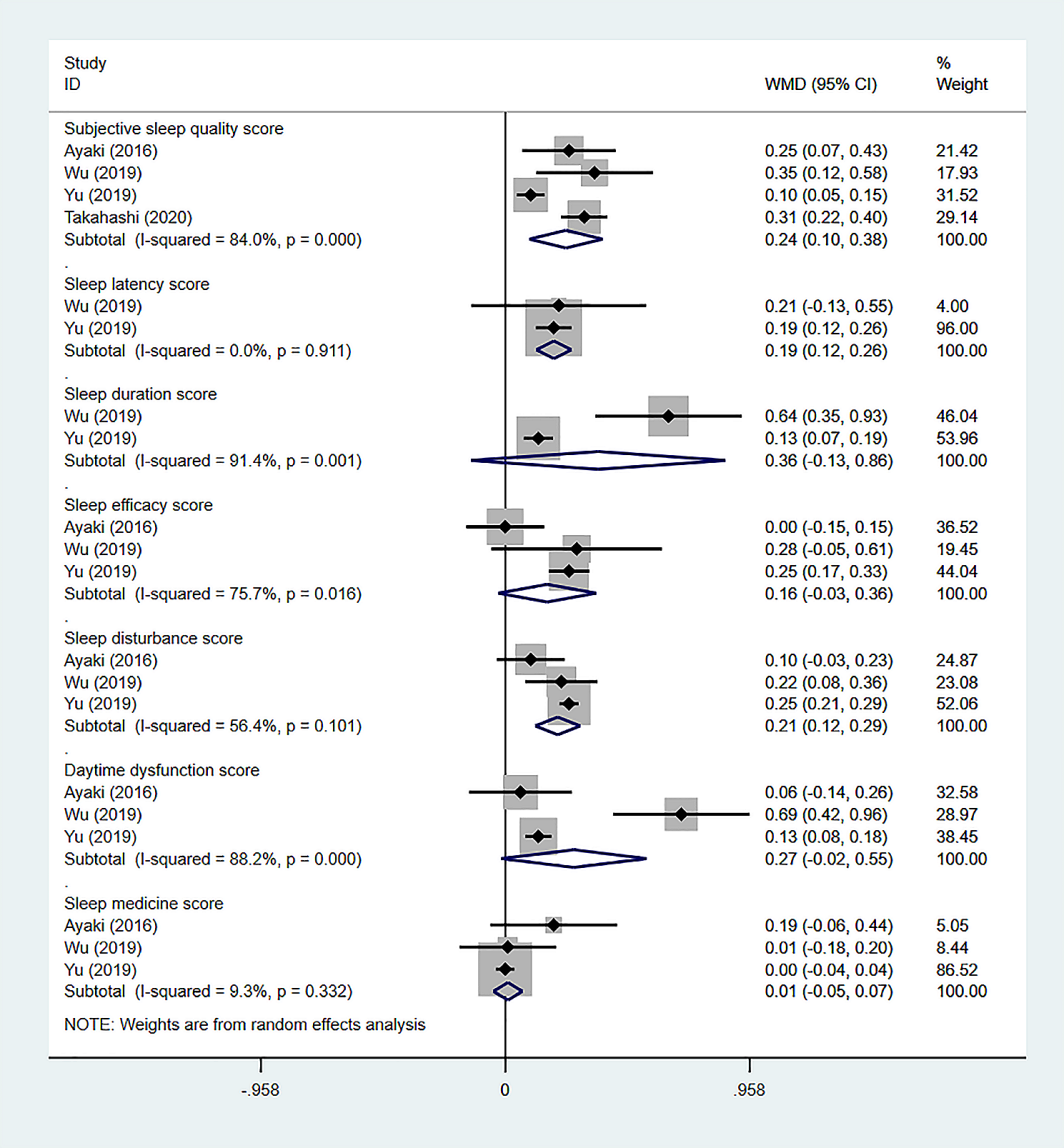
PSQI subitem scores

The PSQI questionnaire scores are divided into subjective sleep quality, sleep latency, sleep duration, habitual sleep efficiency, sleep disturbance, daytime dysfunction, and use of sleep medication. Each domain has a score of 0–3 points. The higher the score, the worse the patient’s performance in that item. A random-effects model was used for the analysis (Fig. 3).

Four articles mentioned the subjective sleep quality scores [14, 27, 30, 31]. The combined results showed that the subjective sleep quality scores of dry eye subjects patients were significantly higher than those of the control subjects (WMD = 0.24, 95%CI: 0.10, 0.38, *P* = 0.001). Two articles mentioned sleep latency scores [30, 31], and the dry eye subjects had significantly higher scores than the control subjects (WMD = 0.19, 95%CI: 0.12, 0.26, *P* < 0.001). Two articles mentioned sleep duration scores [30, 31], and there was no statistically significant difference in the sleep duration scores between the dry eye subjects and the control subjects (WMD = 0.36, 95%CI: -0.13, 0.86, *P* = 0.151). Three articles mentioned sleep efficiency scores [14, 30, 31], and there was no statistically significant difference in the sleep efficiency scores between the dry eye subjects and the control subjects (WMD = 0.16, 95%CI: -0.03, 0.36, *P* = 0.093). Three articles mentioned sleep disturbance scores [14, 30, 31], and the dry eye subjects had significantly higher scores than the control subjects (WMD = 0.21, 95%CI: 0.12, 0.29, *P* < 0.001). Three articles mentioned daytime dysfunction scores [14, 30, 31], and there was no statistically significant difference in the sleep duration scores between the dry eye subjects and the control subjects (WMD = 0.27, 95%CI: -0.02, 0.55, *P* = 0.064). Three articles mentioned used sleep medication scores [14, 30, 31], and there was no statistically significant difference in the sleep medication scores between the dry eye subjects and the control subjects (WMD = 0.01, 95%CI: -0.05, 0.07, *P* = 0.719).

### Sleep disorder

**Fig. 4:**
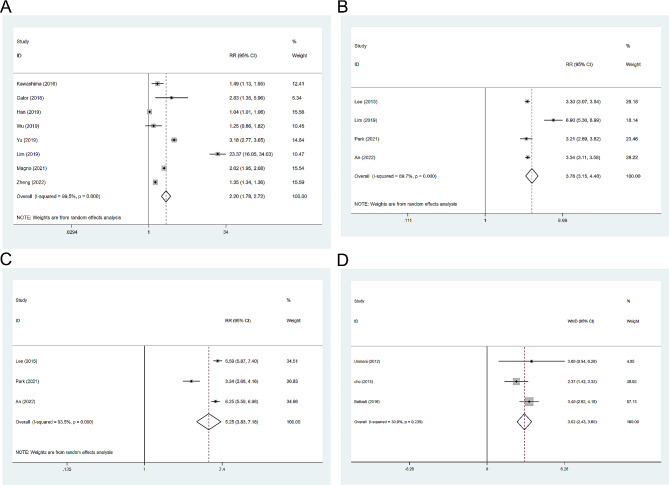
**A**: Number of people with sleep disorders; **B**: Insufficient sleep; **C**: Excessive sleep; **D**: ESS (Epworth Sleepiness Scale) questionnaire;

A total of 8 articles [9, 17, 22, 23, 30–32] reported the number of individuals with sleep disorder (Fig. 4A). After testing for heterogeneity between the subjectss (I^2^ = 99.5%, *P* < 0.001), a random-effects model was used. The results showed that the risk of sleep disorders in the dry eye subjects was significantly higher than in the non-dry eye subjects (RR = 2.20, 95%CI: 1.78, 2.72, *P* < 0.001), with statistically significant differences.

### Insufficient sleep

In this study, sleep duration of less than 5 h was considered insufficient sleep (Fig. 4B). Four articles [8, 21, 22, 25] reported the number of individuals with insufficient sleep (I^2^ = 89.7%, *P* < 0.001), and a random-effects model was used. The results showed that the incidence of insufficient sleep in dry eye patients (RR = 3.76, 95%CI: 3.15, 4.48, *P* < 0.001) was higher than that in the control subjects, with statistically significant differences.

### Excessive sleep

In this study, sleep duration of 9 h or more was considered excessive sleep (Fig. 4C). Three articles [8, 21, 25] reported the number of individuals with excessive sleep based on sleep duration (I^2^ = 93.5%, *P* < 0.001). According to the results, the incidence of excessive sleep in dry eye patients was higher than that in the control subjects (RR = 5.53, 95%CI: 3.83, 7.18, *P* < 0.001), with statistically significant differences.

Some articles [15, 16, 28] also used the ESS (Epworth Sleepiness Scale) questionnaire to investigate the likelihood of daytime sleepiness (Fig. 4D). The ESS questionnaire consists of 8 questions, mainly about the patient’s typical daytime sleepiness situation [16]. Cho et al. [16] concluded that dry eye patients had higher ESS scores and were more likely to experience daytime sleepiness. According to the analysis, dry eye patients are more likely to experience excessive sleepiness (WMD = 3.02, 95%CI: 2.43, 3.60, *P* < 0.01), with statistically significant differences.

### Other Sleep-Related scales

Gudbjörnsson et al. [18]used the Uppsala Sleep Inventory to assess sleep conditions, which is an 87-item survey questionnaire completed by the patients themselves. Patients’ performances in the questionnaire were divided into five levels (five-point scale). Gudbjörnsson et al. [18] chose the top two levels of severity for analysis, namely “’great”/“very great” and " often “/“ very often”. The results showed that patients with pSS (primary Sjogren’s syndrome) had more tense muscles when falling asleep, and experienced anxiety and racing thoughts compared to healthy individuals. At the same time, they were more likely to wake up at night and feel more tired during the day.

The ISI (Insomnia Severity Index) questionnaire was also included in the literature of this study to assess patients’ insomnia [17]. Galor et al. [17] found that the ISI scores of dry eye patients (13.3 ± 8.3) were significantly higher than those of non-dry eye patients (7.3 ± 7.3), suggesting that dry eye patients are more likely to suffer from insomnia. Additionally, the severity of eye pain symptoms was related to the severity of insomnia. Lim et al. [22] observed that there was a significant association between dry eye and insomnia (OR = 1.08, 95%CI: 1.05, 1.11, *P* < 0.001).

## Discussion

Compared to previously published systematic reviews, our analysis only included healthy controls and analyzed 22 studies involving 419,218 patients to draw conclusions. Current research results show that the sleep quality of dry eye patients is significantly worse than that of the healthy population, and the risk of sleep disorders is significantly higher than that of the normal population. Based on PSQI questionnaire scores, dry eye patients have poorer subjective sleep quality, longer sleep latency, and have sleep disorders; however, there is no difference in sleep duration, sleep efficiency, daytime dysfunction, or use of sleeping medications. Dry eye patients are more likely than healthy individuals to have the risk of unhealthy sleep duration, such as insufficient sleep or excessive sleep.

Our literature review confirms the previous conclusion by Au et al. [10]that dry eye patients are more likely to have sleep disorder. However, there is a slight discrepancy regarding whether dry eye patients are more prone to daytime issues. Au et al. [10] believed that dry eye patients are more likely to have daytime sleepiness, while we found no difference between dry eye patients and the healthy population in terms of daytime dysfunction. This is related to our different definitions of daytime behavior. Besides, Au et al.‘s [10]systematic review included control subjectss with other eye diseases, while our control subjects consisted only of healthy individuals. There are some contradictions in the conclusions of this study. Based on the PSQI questionnaire, there is no difference in total sleep duration between dry eye patients and healthy people, but dry eye patients are more likely to have extreme sleep duration according to statistics. This may be related to an increased sample size or the subjectivity of the questionnaire.

Poor sleep qualities in dry eye patients may be due to light exposure and discomfort caused by incomplete eyelid closure or pain caused by inflammation [8]. Dry mouth discomfort experienced by dry eye and pSS patients is also a contributing factor. At the same time, dry eye patients are more likely to suffer from anxiety and depression [33], with a prevalence of about 29%, making it one of the eye diseases most likely to cause depression [34]. Emotional disorders, such as stress, can also cause sleep disturbance. In a study by Ayaki et al. [35], dry eye patients were provided with treatments including eye drops, eye care, nutritional supplements, and daily advice. The patients’ PSQI scores decreased, and their sleep quality improved, further supporting the findings of this study.

Additionally, there is a bidirectional association between dry eye and sleep disorders; poor sleep or sleep deprivation may also lead to the onset or exacerbation of dry eye symptoms. An intervention study by Lee et al. [36] showed that sleep deprivation (SD) induces increased tear osmolarity, shortened tear film break-up time, and reduced tear secretion, thereby further triggering the onset and development of dry eye. Physiologically, sleep disorders often lead to autonomic nervous system dysfunction, affecting the parasympathetic function in the lacrimal gland and reducing tear secretion [31, 37].

Although this study tried to include high-quality studies from different countries, there are still some limitations. Firstly, most of the included articles used questionnaires to determine whether patients had dry eye or sleep disorders, and questionnaires have a certain degree of subjectivity, with different respondents having different standards. Secondly, most of the included articles were cross-sectional studies, with little discussion on the mechanisms and causal relationships underlying the increased incidence of sleep disorders in dry eye patients. Finally, dry eye is more common in women and elderly patients, and the original studies included in this study adjusted for the gender factor, leading to the inclusion of more elderly women in the study population compared to other subjects.

## Conclusion

Our meta-analysis indicates that dry eye patients have a lower sleep quality than the healthy population, with poorer subjective sleep quality, longer sleep latency, and a higher risk of unhealthy sleep duration such as insufficient sleep or excessive sleep.

However, so far, there is not enough evidence to establish a causal relationship and related mechanisms between dry eye and sleep disorder. In the future, more large-scale prospective studies are needed to provide more assistance in patient management and treatment.

### Electronic supplementary material

Below is the link to the electronic supplementary material.

Supplementary Material 1: Search Strategy

## Data Availability

The data that support the findings of this study are available from the corresponding author upon reasonable request.
