# Supplementary material for: Association between sleep quality and dry eye disease: a literature review and meta-analysis
Source: BMC Ophthalmol. 2024 Apr 5;24:152. doi: 10.1186/s12886-024-03416-7 (PMC10996221; doi:10.1186/s12886-024-03416-7)
Supplement: Supplementary file 1 — Supplementary Material 1: Search Strategy [file 12886_2024_3416_MOESM1_ESM.docx]

**Supplementary material 1** Search Strategy

**1. Pubmed**

1 (((((sleep*[Title/Abstract]) OR (insomnia*[Title/Abstract])) OR (dyssomnia*[Title/Abstract])) OR (DIMS[Title/Abstract])) OR (awake*[Title/Abstract])) OR (sleep disturbance*[Title/Abstract])

2 (((((dry eye*) OR (dysfunctional tear syndrome*)) OR (Sicca)) OR (Sjogrens)) OR (Sjögren's)) OR (Sjogren)

3 (("Sleep"[Mesh]) OR "Sleep Initiation and Maintenance Disorders"[Mesh]) OR "Dyssomnias"[Mesh]

4 ("Dry Eye Syndromes"[Mesh]) OR "Sjogren's Syndrome"[Mesh]

5 (("Dry Eye Syndromes"[Mesh]) OR "Sjogren's Syndrome"[Mesh]) OR ((((((dry eye*) OR (dysfunctional tear syndrome*)) OR (Sicca)) OR (Sjogrens)) OR (Sjögren's)) OR (Sjogren))

6 ((("Sleep"[Mesh]) OR "Sleep Initiation and Maintenance Disorders"[Mesh]) OR "Dyssomnias"[Mesh]) OR ((((((sleep*[Title/Abstract]) OR (insomnia*[Title/Abstract])) OR (dyssomnia*[Title/Abstract])) OR (DIMS[Title/Abstract])) OR (awake*[Title/Abstract])) OR (sleep disturbance*[Title/Abstract]))

7 ((("Sleep"[Mesh]) OR "Sleep Initiation and Maintenance Disorders"[Mesh]) OR "Dyssomnias"[Mesh]) OR ((((((sleep*[Title/Abstract]) OR (insomnia*[Title/Abstract])) OR (dyssomnia*[Title/Abstract])) OR (DIMS[Title/Abstract])) OR (awake*[Title/Abstract])) OR (sleep disturbance*[Title/Abstract]))

**2. Cochrane**

1. MeSH descriptor: [Sleep] explode all trees
2. MeSH descriptor: [Sleep Initiation and Maintenance Disorders] explode all trees
3. MeSH descriptor: [Dyssomnias] explode all trees
4. MeSH descriptor: [Dry Eye Syndromes] explode all trees
5. MeSH descriptor: [Sjogren's Syndrome] explode all trees
6. (sleep*):ti,ab,kw OR (insomnia*):ti,ab,kw OR (dyssomnia*):ti,ab,kw OR (awake*):ti,ab,kw OR (sleep disturbance*):ti,ab,kw
7. (dry eye*):ti,ab,kw OR (dysfunctional tear syndrome*):ti,ab,kw OR (Sicca):ti,ab,kw OR (Sjogrens):ti,ab,kw OR (Sjögren's):ti,ab,kw
8. (DIMS):ti,ab,kw
9. (Sjogren):ti,ab,kw
10. #1 OR #2 OR #3 OR #6 OR #8
11. #4 OR #5 OR #7 OR #9
12. #10 AND #11

**3. Embase**

1 'sleep disorder*':ab,ti OR sleep*:ab,ti OR insomnia*:ab,ti OR dyssomnia*:ab,ti OR awake*:ab,ti OR 'sleep disturbance*':ab,ti OR dims:ab,ti

2 'sleep'/exp OR 'sleep disorder'/exp

3 'dry eye'/exp OR 'dry eye syndrome'/exp OR 'sjoegren syndrome'/exp

4 'dry eye*':ab,ti OR sicca:ab,ti OR sjogrens:ab,ti OR sj grens:ab,ti OR sjogren:ab,ti OR 'dry eye syndrome*':ab,ti OR 'dysfunctional tear syndrome*':ab,ti

5 1 OR 2

6 3 OR 4

7 5 AND 6

**4. Web of Science**

**1 sleep*** (Topic) or **Insomnia*** (Topic) or **Awake*** (Topic) or **Dyssomnia*** (Topic) or **DIMS** (Topic) or **sleep disturbance*** (Topic)

**2 dry eye*** (Topic) or **dysfunctional tear syndrome*** (Topic) or **Sicca** (Topic) or **Sjogrens** (Topic) or **Sjogren** (Topic) or **Sjögren's** (Topic) or **Sjögren's syndrome** (Topic)

3 1 AND 2
